# The Next Frontier in Brain Monitoring: A Comprehensive Look at In-Ear EEG Electrodes and Their Applications

**DOI:** 10.3390/s25113321

**Published:** 2025-05-25

**Authors:** Alexandra Stefania Mihai (Ungureanu), Oana Geman, Roxana Toderean, Lucas Miron, Sara SharghiLavan

**Affiliations:** 1 Computers, Electronics and Automation Department, Faculty of Electrical Engineering and Computer Science, Stefan cel Mare University of Suceava, 720229 Suceava, Romania; stefania.mihai1@student.usv.ro (A.S.M.); roxana.toderean@usm.ro (R.T.); lucas.miron@student.usv.ro (L.M.); sara.sharghi2020@gmail.com (S.S.); 2 Data Science and AI Group, Department of Computer Science Engineering, Chalmers University of Technology, 412 96 Gothenburg, Sweden; 3 Data Science and AI Group, Department of Computer Science Engineering, University of Gothenburg, 405 30 Gothenburg, Sweden; 4 Department of Cognitive Neuroscience, Faculty of Education and Psychology, University of Tabriz, Tabriz 51666-16471, Iran

**Keywords:** electroencephalogram (EEG), EEG evolution, electrodes, EEG signal, portable devices, miniaturization, contact impedance

## Abstract

Electroencephalography (EEG) remains an essential method for monitoring brain activity, but the limitations of conventional systems due to the complexity of installation and lack of portability have led to the introduction and development of in-ear EEG technology. In-ear EEG is an emerging method of recording electrical activity in the brain and is an innovative concept that offers multiple advantages both from the point of view of the device itself, which is easily portable, and from the user’s point of view, who is more comfortable with it, even in long-term use. One of the fundamental components of this type of device is the electrodes used to capture the EEG signal. This innovative method allows bioelectrical signals to be captured through electrodes integrated into an earpiece, offering significant advantages in terms of comfort, portability, and accessibility. Recent studies have demonstrated that in-ear EEG can record signals qualitatively comparable to scalp EEG, with an optimized signal-to-noise ratio and improved electrode stability. Furthermore, this review provides a comparative synthesis of performance parameters such as signal-to-noise ratio (SNR), common-mode rejection ratio (CMRR), signal amplitude, and comfort, highlighting the strengths and limitations of in-ear EEG systems relative to conventional scalp EEG. This study also introduces a visual model outlining the stages of technological development for in-ear EEG, from initial research to clinical and commercial deployment. Particular attention is given to current innovations in electrode materials and design strategies aimed at balancing biocompatibility, signal fidelity, and anatomical adaptability. This article analyzes the evolution of EEG in the ear, briefly presents the comparative aspects of EEG—EEG in the ear from the perspective of the electrodes used, highlighting the advantages and challenges of using this new technology. It also discusses aspects related to the electrodes used in EEG in the ear: types of electrodes used in EEG in the ear, improvement of contact impedance, and adaptability to the anatomical variability of the ear canal. A comparative analysis of electrode performance in terms of signal quality, long-term stability, and compatibility with use in daily life was also performed. The integration of intra-auricular EEG in wearable devices opens new perspectives for clinical applications, including sleep monitoring, epilepsy diagnosis, and brain–computer interfaces. This study highlights the challenges and prospects in the development of in-ear EEG electrodes, with a focus on integration into wearable devices and the use of biocompatible materials to improve durability and enhance user comfort. Despite its considerable potential, the widespread deployment of in-ear EEG faces challenges such as anatomical variability of the ear canal, optimization of ergonomics, and reduction in motion artifacts. Future research aims to improve device design for long-term monitoring, integrate advanced signal processing algorithms, and explore applications in neurorehabilitation and early diagnosis of neurodegenerative diseases.

## 1. Introduction

Electroencephalography is the most effective and widespread reference method for recording electrical activity in the brain, performed by placing surface electrodes on the scalp. Classical EEG recording involves the use of a variable number of electrodes, depending on the specific application. Thus, clinical EEG typically uses 19, 32, or even 64 electrodes, while high-density EEG systems may include 128 or 256 electrodes. These electrodes are accompanied by a common reference electrode, which is placed in electrically neutral regions such as the earlobe, the base of the nose, or the nasal fossa [1].

The classical method of EEG recording provides valuable information, but its use is accompanied by multiple challenges. The conventional EEG system involves a complex network of wires connecting the electrodes attached to the scalp to the amplifier, which significantly complicates the procedure. The installation process is also time-consuming and must be carried out by trained personnel, requiring specialized hardware and a controlled recording frame. Therefore, despite its unique and undeniable usefulness, the classical way of EEG recording proves to be complicated and cumbersome. These limitations led, in 2011 [2], to the introduction of a new concept of EEG recording, known as in-ear EEG.

**Figure 1:**
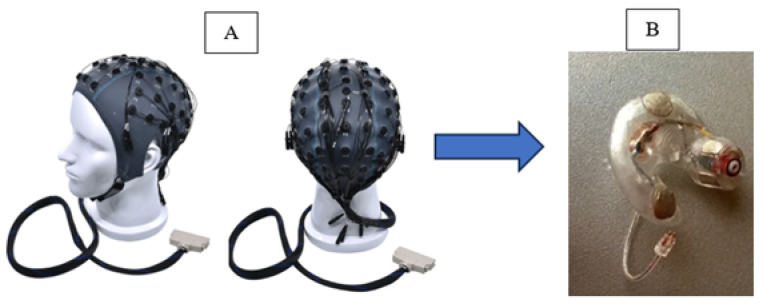
(**A**) Conventional EEG collection system and (**B**) wearable in-ear EEG device with embedded electrodes.

The evolving EEG landscape is taking shape by integrating this type of recording into a highly promising emerging innovation. In-ear EEG is proposed and materialized in a minimally invasive, hearing aid-like solution (Figure 1B). The acquisition of EEG signals with these ergonomic in-ear devices with embedded electrodes offers the advantage of portability, comfort, and long-term wearability [3]. In contrast to classical EEG methods using electrodes placed on the scalp, in-ear EEG can be used unobtrusively even during everyday activities, which makes it particularly attractive for cognitive research, neurodiagnostics, and consumer health.

Due to its unique advantages, in-ear EEG has generated notable interest as an alternative or complement to traditional and wearable EEG technologies, thus striking a balance between clinical accuracy and usability in everyday life. The in-ear EEG approach is preferred over other methods due to its potential applications in clinical diagnostics, cognitive research, and especially in settings where conventional methods cannot be implemented or need to be invasive [4,5,6].

The use of in-ear EEG in the wider electroencephalographic landscape represents a major shift in neuronal activity monitoring, offering a perfect combination of portability, unobtrusiveness, and continuous and adequate data acquisition. The use of conventional EEG is cumbersome due to the very bulky setups that require coverage of the entire scalp, thus reducing the possibility of its use outside of less controlled environments, such as during the patient’s daily activities.

Technically, intra-auricular EEG takes advantage of the unique anatomical and electrophysiological properties of the ear, where the restricted space and stable positioning contribute to reduced motion artifacts and improved signal fidelity. This new recording model has also seen and spurred advances in customized electrode materials, including PEDOT:PSS (poly(3,4-ethylenedioxythiophene)/polystyrene sulfonate), a conductive polymer widely used in bioelectronics for its high conductivity and biocompatibility; graphene derivatives; and conductive hydrogels, all aimed at optimizing conductivity while maintaining comfort and biocompatibility. Recent work by Gao et al. introduced a pin-shaped Ag/AgCl textile electrode coated with a self-hydrating hydrogel, which achieved low contact impedance and excellent signal fidelity in hairy scalp regions, demonstrating the potential of hybrid material designs for improving EEG performance in challenging recording conditions [7]. In the wider EEG ecosystem, the in-ear EEG is poised to complement and, in certain applications, replace the classical scalp EEG, especially in areas that require continuous neuronal monitoring under real-world conditions. Its integration into brain–computer interfaces (BCIs), cognitive monitoring, and clinical neurodiagnostics supports the development of neurophysiological assessment tools that are discreet, non-invasive, and suitable for daily-life use.

Despite these remarkable advances, challenges remain, particularly in terms of spatial resolution, inter-individual anatomical variability, and the development of advanced signal processing techniques to mitigate the inherent limitations of a compact electrode array. However, due to continued progress in artifact reduction through artificial intelligence, sensor miniaturization, and multimodal integration, in-ear EEG is emerging as a transformative innovation, redefining the trajectory of non-invasive brain activity monitoring.

To address all these challenges, the field of mobile EEG has evolved significantly, fostering the development of alternative electrode configurations and recording methods that emphasize usability, portability, and real-world applicability.

## 2. History and Evolutionary Steps of EEG in the Ear

Recent studies shed new light on the role of the ear in EEG data acquisition. Information on brain activity, cardiovascular, respiratory, and motor functions can be collected from the ear. Thus, the ear would enable the development of health monitoring devices with miniaturized, high-discretion, miniaturized sensors that also exhibit robustness to artifacts and noise [8].

The basis of these studies is the anatomical perspective of the ear. Anatomically, the ears—paired organs located on either side of the skull—are responsible for the sense of hearing. A true sound conduction and transmission mechanism has developed in the ear, divided into two main stages, depending on their localization. Thus, the outer ear has the role of capturing sound, while impedance matching is performed in the middle ear. From an EEG perspective, the anatomy of the ear is not only relevant for sound conduction but also for the optimal positioning of electrodes. The anatomical configuration of the ear plays an essential role in the design of intra-aural EEG devices. Beyond its function in transmitting acoustic waves, the structure of the ear—especially the outer and middle ear—allows for acoustic impedance matching, facilitating efficient energy transfer. The same architecture also provides stable and morphologically favorable contact areas for electrode placement. As a result, in-ear EEG systems benefit from improved signal quality and reduced motion artifacts due to the natural shape and impedance characteristics of the ear canal. These anatomical considerations are an important starting point in selecting the optimal materials and configuration for electrodes integrated into intra-aural devices.

The external auditory canal from the pinna to the eardrum is generally 26 mm long and 7 mm in diameter. These dimensions have been considered in the design of ear-canal phones. In [2], the designed device does not penetrate more than 10 mm into the ear, does not localize in the bone surrounding part of the ear canal, and does not affect the eardrum in any way. From an electrophysiologic perspective, the cortex exhibits bioelectrical signals that are attenuated by the cerebrospinal fluid, skull, and skin before these signals reach the ear canal. This attenuation of bioelectrical signals is similar to conventional scalp measurements using classical EEG [8].

**Figure 2:**
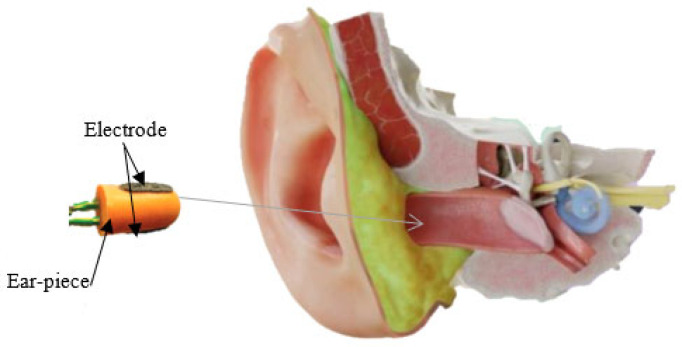
In-ear EEG device with embedded electrodes in the ear canal.

This new approach, EEG in the ear, has been highlighted by Looney D. and his collaborators, who propose the placement of electrodes inside a specific hearing protection customized in-ear EEG device, which will then be localized in the ear canal (Figure 2) [2]. The earmold-based EEG system thus used, approved, and customized proved to be more comfortable compared to the classical EEG. This device could be used for recordings in optimal conditions much easier, even among children [3].

**Figure 3:**
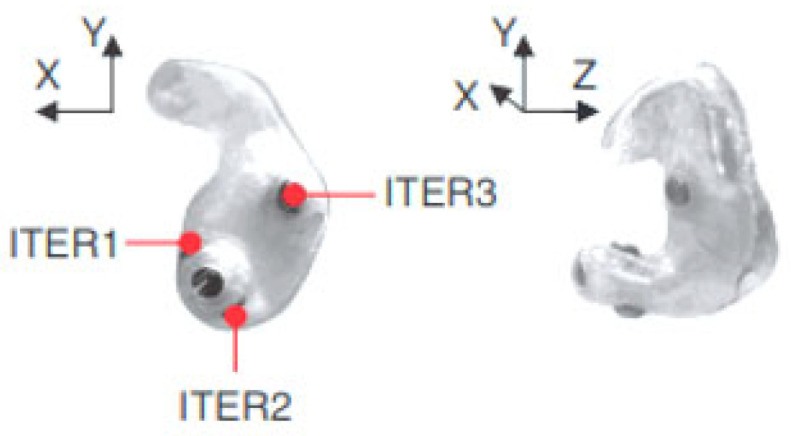
Configuration of the right ear EEG recording system in the right ear in different anatomical planes (IREL). Adapted from [9].

The earpiece with embedded electrodes used for EEG recordings in the ear incorporates electrodes that are made of silver chloride (AgCl), a material recognized as the gold standard in electrode manufacturing for this type of application. Each earmold is designed to anatomically fit each ear with a specific configuration, illustrated as an example for the right ear in Figure 3. In this regard, the earplug is equipped with three electrodes—ITEL1, ITEL2, and ITEL3—strategically arranged in different anatomical planes. These electrodes are integrated into the structure of the plug intended for insertion into the ear canal, ensuring optimal contact for capturing EEG signals.

Thus, the portable in-ear EEG system offers multiple advantages, including high comfort, accessibility for the patient, and the possibility to carry out daily activities in a normal manner, free from the constraints imposed by EEG recording. Meanwhile, the acquisition of electroencephalographic data is performed continuously, the starting point being a simplified installation process that does not require laborious procedures, as well as the stability of electrode positioning, which contributes significantly to improving the repeatability of experiments and obtaining reliable data [3].

To evaluate the performance of the electrodes integrated into the intra-auricular plug and to be able to validate the EEG recordings made in the ear, Looney et al. performed comparative tests using a conventional EEG system with scalp-mounted electrodes. This study was performed on one participant using the recording setup shown in Figure 3. Analysis of the EEG data revealed that the electrodes placed in the ear recorded a lower amplitude signal compared to that obtained with scalp electrodes. At the same time, the recorded noise level was also lower, which led to a similarity between the signal-to-noise ratio for the two methods of EEG signal acquisition.

In conclusion, a study that introduces for the first time the concept of EEG in the ear validates the feasibility of this innovative method of electroencephalographic recording, based on the use of electrodes inserted in the external auditory canal. The results demonstrated a correlation between the signals picked up at the intra-auricular level and those recorded at the scalp, which has subsequently provided the basis for numerous research directions. This technology is characterized by its non-invasiveness, high esthetic acceptability, and the possibility of both short-term and long-term monitoring. In-ear EEG thus represents a promising solution for continuous assessment of brain activity under natural conditions.

In terms of chronological trajectory, several key moments in the evolution of the concept of EEG in the ear are remarkable: the introduction of the concept and the first experimental studies described above were just the starting point of a remarkable ascent. This was followed by the development and optimization of the materials from which the electrodes used in in-ear EEG devices today are made. Among these materials are AgCl electrodes—also a standard in today’s configurations—which play a role in improving electrical conductivity and reducing artifacts.

With the introduction of the new concept and the development of the component materials, the first devices to improve the original equipment also appeared. This continuous process of evolution persists to the present day, with the goal of integrating in-ear EEG into wearable devices and the possibility of continuous health monitoring. Although customized earpieces may involve higher initial costs, the process is already well-established in the hearing aid and earplug industry, with affordable and scalable production methods. Custom-molded designs offer superior comfort, better mechanical stability, and improved electrode–skin contact, which directly impact signal quality and motion robustness. On the other hand, generic earpieces are easier to mass-produce and suitable for short-term or population-level deployments, albeit with slightly reduced signal consistency due to anatomical variability. The aim is to achieve recordings as close as possible to classical EEG. A new idea is proposed to support the concept, namely the use of generic in-ear devices. The customization of earmold-based EEG systems according to the anatomical dimensions of each patient’s ear would be a laborious option involving a multi-step process: wax impression of the outer ear of each individual patient, subsequent 3D scanning of the ear impression, modeling of the ear impression in various software programs, and then manufacturing the in-ear piece. Hence, the process would be costly and time-consuming. Thus, a prototype with a customized in-ear EEG device made of silicone rubber as its core was tested. According to [5], “comparative analysis between customized and generic prototypes provides insight into how electrode positioning affects the recording quality in in-ear EEG devices”.

**Figure 4:**
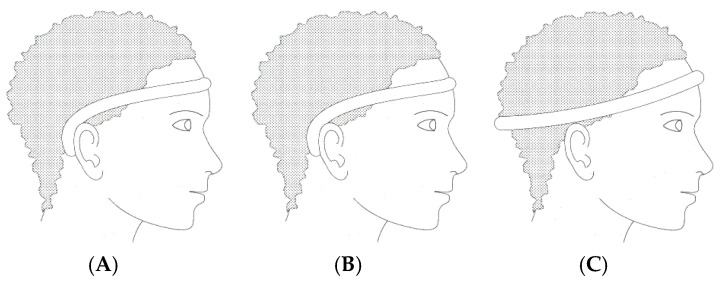
Different types of EEG recording headbands: (**A**) frontal, (**B**) near, and (**C**) full circle.

In recent years, various portable EEG modalities have been developed to improve comfort, mobility, and real-world usability compared to conventional scalp-based systems. These include wearable headbands with dry electrodes, periauricular devices positioned around the ear, tattoo-based flexible electrodes, and clip-on ear systems. Among these, in-ear EEG has emerged as a distinct and promising direction, offering a unique balance between signal fidelity and user comfort by integrating electrodes directly into the ear canal. While this review focuses primarily on in-ear EEG systems, it is important to consider them in the broader context of portable EEG technologies, where trade-offs exist between signal quality, ease of use, and degree of invasiveness. Further research directions have also focused on the use and integration of EEG in devices such as EEG headbands with dry electrodes (Figure 4), which eliminate the need for conductive gels while maintaining adequate signal fidelity.

**Figure 5:**
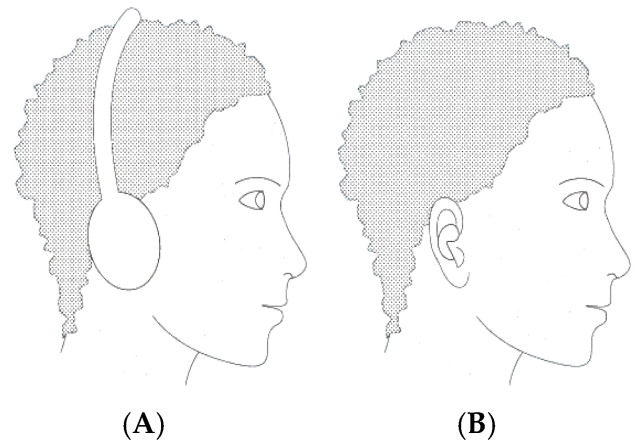
(**A**) Periauricular system for EEG recording. (**B**) Intra-auricular device for EEG recording.

At the same time, periauricular EEG systems (Figure 5A) are also found, which position the electrodes around the ear, optimizing stability and reducing motion artifacts.

**Figure 6:**
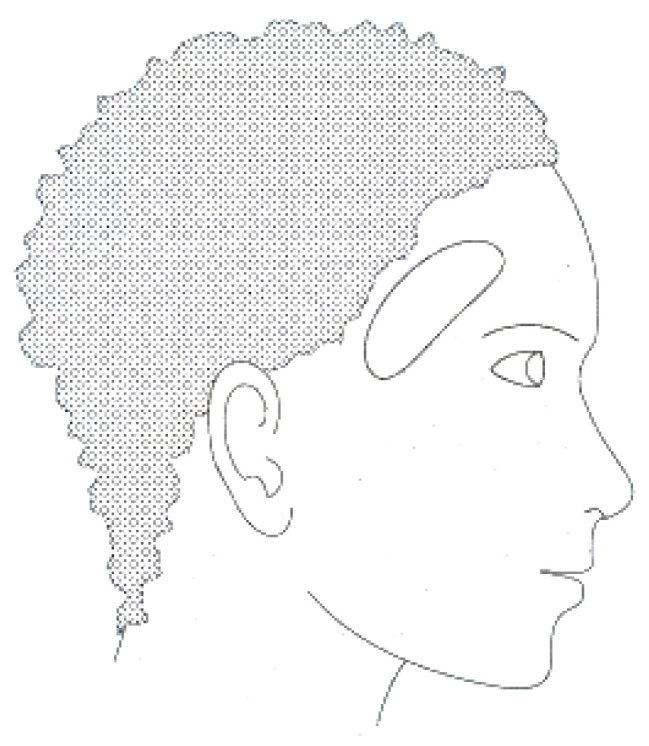
Adhesive device for EEG recording.

Tattoo-shaped EEG stickers (Figure 6), made of ultra-thin, flexible substrates that adhere to the skin for discreet and prolonged monitoring, are also reliable recording options. Practical and easy solutions are also offered by clip-type ear electrodes, which use the auricle and mastoid as stable recording points. All these new variants support the evolution and integration of the concept introduced by Looney for easier patient health monitoring.

This review focuses specifically on in-ear EEG while acknowledging its position within the broader landscape of portable EEG technologies. This comparative framework highlights the position of in-ear EEG within the evolving field of wearable neurotechnologies, where its advantages in comfort and discreetness make it particularly suited for continuous and ambulatory brain monitoring.

## 3. Definition and Conceptual Delimitation

In the literature, the terms in-ear EEG (in-ear EEG, in-ear EEG, and auricular EEG) are often used with different meanings, which can lead to conceptual and methodological confusion. To clearly outline the scope of analysis, in this paper we propose the following classification (see Table 1):

We note that although some papers use the term ‘in-ear EEG’ for all these forms, in this review we focus mainly on intra-auricular devices, with some complementary references to periauricular systems when contributing to the understanding of technological design or clinical applications.

In the remainder of this review, the term in-ear EEG will be used consistently to refer to intra-auricular systems, in accordance with the classification provided in this section.

## 4. Comparative Aspects of Conventional EEG—In-Ear EEG

Both conventional EEG and in-ear EEG are methods for recording brain bioelectrical activity, but there are notable differences between the two approaches in terms of the technology used, the accuracy of the measurements, and their applicability in different contexts.

### 4.1. Technical Characteristics of In-Ear EEG: Comparison with Conventional EEG

In-ear EEG technology has been proposed as a portable, minimalist, and unobtrusive alternative to conventional EEG systems, offering advantages in terms of user comfort and applicability in natural contexts. However, the performance of these systems is influenced by specific technical factors, which deserve to be systematically analyzed to understand the real potential and limitations of the technology.

### 4.2. Architecture of In-Ear EEG Systems

In-ear EEG systems generally use a small number of electrodes (between 2 and 6) placed in the external auditory canal (intra-auricular) or periauricular area. This electrode distribution limits the recording capability to brain regions adjacent to the auricular area [10,11]. The in-ear EEG configurations described in [11,12] confirm the use of a reduced number of electrodes, thus affecting the spatial resolution of the measurements. This limited number of electrodes is not only a design choice but also a physical constraint imposed by the confined anatomical space of the ear canal. The need to ensure comfort, mechanical stability, and effective skin contact restricts the possibility of increasing electrode density. This creates an inherent bottleneck in spatial resolution, as denser electrode arrays—common in high-resolution scalp EEG—are not feasible in this setting. Innovative solutions such as microfabricated electrodes, layered electrode architectures, or multiplexed contact points may offer future ways to circumvent this limitation. The electrodes used in in-ear EEG devices can be dry (made of solid conductive materials such as gold or silver) or wet (with conductive gels). The choice of configuration depends on the application: research systems favor higher fidelity, while wearable applications are oriented towards ergonomics and ease of use.

### 4.3. Performance Parameters: Comparison with Conventional EEG

Compared to conventional EEG, in-ear EEG systems show significant differences in signal quality and nature. Parameters such as interface impedance, signal amplitude, signal-to-noise ratio (SNR), or susceptibility to artifacts vary significantly between the two approaches. For a structured interpretation, Table 2 presents a thematic comparison between conventional EEG and in-ear EEG, organized into four categories: electrode type, acquisition method, signal quality parameters, and ergonomic aspects.

To highlight the differences between conventional EEG and in-ear EEG in terms of key functional parameters (number of electrodes, placement, signal quality, amplitude, comfort, artifacts, installation, and portability), the table below provides a comparative graphical representation (Table 3). Qualitative markers are used (++ high, + medium, − low), based on a synthesis of trends consistently reported in the literature.

From Table 3, we can conclude that conventional EEG typically achieves higher signal amplitude and SNR values, making it more appropriate for clinical precision applications. In contrast, in-ear EEG offers superior comfort and portability, making it suitable for continuous, real-life monitoring scenarios.

### 4.4. Technical Limitations and Challenges

One of the main limitations of EEG in the ear is the high impedance of the electrode-electrode interface, especially in the absence of conductive gel. This leads to decreases in signal-to-noise ratios and in signal amplitudes, which can be up to ten times smaller than in conventional EEGs. Spatial resolution is also limited due to the small number of channels and limited coverage of scalp areas. On the other hand, motion artifacts are also more frequent in recordings performed outside the laboratory, which require advanced processing of the acquired signal. These methods will be discussed in more detail in the section dedicated to intra-auricular EEG signal processing [5].

### 4.5. Artifact Reduction and EEG Signal Processing

Artifact reduction in intra-auricular electroencephalography is particularly important for improving neural signal quality. Although EEG is a well-established method for measuring electrical activity in the brain, its efficiency is quite often compromised by various artifacts that can obscure genuine neuronal signals. The artifacts, depending on the origin of the source that generates them, can be classified into two main categories: physiological—products of normal biological activity of the organism (for example, eye movements and muscular activity). The second class of artifacts are non-physiological artifacts that are produced by external sources, which are not related to the human organism itself but can be generated by various technical malfunctions of the recording equipment, e.g., electrical interference from external sources. The presence of artifacts only hinders the process of interpretation and analysis of the acquired data [13,14,15]. Motion artifacts are particularly relevant in in-ear EEG because the ear canal is directly affected by jaw and facial muscle movements such as chewing, speaking, or smiling. These actions can disturb the stability of electrode contact or induce mechanical shifts in the device, causing transient signal fluctuations. In contrast, scalp EEG setups typically rely on fixed electrode caps that offer more stable positioning. The importance of effectively managing artifacts in EEG recordings has led to the development of advanced techniques, including independent component analysis (ICA), empirical mode decomposition (EMD), and filtering methods [16,17,18]. The above strategies are designed to identify and eliminate unwanted noise. Simultaneously, however, the preservation of underlying neural signals is considered to improve the integrity of the data obtained during the experiments. In particular, the integration of ICA and EMD has shown promise in significantly improving artifact reduction capabilities, allowing more accurate analysis of cognitive processes and clinical conditions [19].

Independent component analysis (ICA) is a method often used to separate mixed signals recorded in EEG data. ICA consists of the identification and subsequent isolation of independent components. In this manner researchers can effectively eliminate unwanted artifacts such as physiological artifacts. The steps taken in performing the ICA technique consist of creating a remixing matrix and then separating the independent components that are analyzed to identify associated artifacts [18,20].

Improving the quality of the electroencephalographic signal can also be achieved by means of the Empirical Mode Decomposition (EMD) method. Using EMD, the obtained signal is decomposed into intrinsic mode functions. This allows selective noise removal while preserving the underlying neural signals. ICA and EMD are therefore methods that, when combined, significantly enhance the improvement of strategies to reduce artifact from EEG signal acquisition [19].

Another powerful processing alternative is wavelet transforms, which offer the advantage of analyzing the EEG signal in the time-frequency domain. This approach allows for the peculiar identification of transient artifacts, such as rapid muscle contractions or head movements, by analyzing frequency scales. The use of discrete wavelets allows adaptive denoising while maintaining the relevant brain signal components [21].

Recently, artificial intelligence-based methods, in particular deep neural networks, have gained ground in real-time EEG processing. Convolutional networks (CNNs) can be trained to recognize artifact patterns directly from the raw signal, without requiring prior filtering steps. Long Short-Term Memory (LSTM) models can learn some complex temporal sequences and are used in context-dependent artifact detection. These methods offer potential for direct integration of in-ear EEG directly into wearable systems for automated real-time use [22]. The use of these advanced methods in wearable in-ear EEG systems leads to increased robustness, helping to create reliable applications in cognitive neuroscience, continuous medical monitoring, and brain–computer interfaces. In particular, the combination of classical techniques (ICA and wavelet) and hybrid AI models provides a balance between interpretability, accuracy, and speed of execution [23].

## 5. Technical Considerations of Electrodes Used in In-Ear EEG

### 5.1. Types of Electrodes Used in EEG in the Ear: Design and Configuration

The evolution of EEG in the ear brings with it the evolution of the types of electrodes used. The design of in-ear EEG systems mainly involves the integration of small metal disks—electrodes—designed to capture electrical signals generated by neural activity. This technology capitalizes on recent advances in materials, aiming to both optimize user comfort and improve signal quality [3].

**Figure 7:**
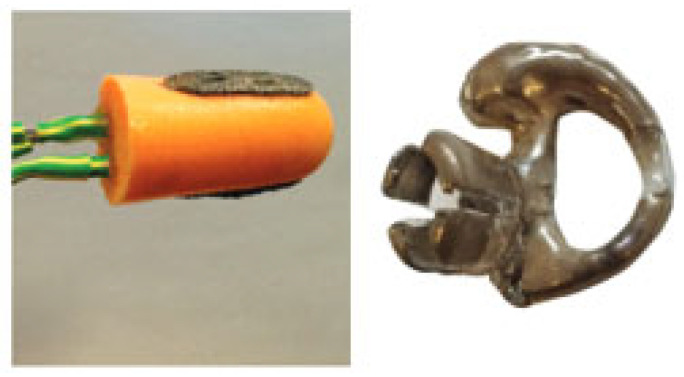
In-ear EEG using wet and dry electrodes, respectively. Adapted from [24,25].

Two broad classes of electrodes used in intra-auricular EEG recording devices have been differentiated: wet electrodes and dry electrodes (Figure 7).

#### 5.1.1. A Wet Electrodes

The first electrodes used in the development of the first in-ear EEG prototype were made of silver/silver chloride (Ag/AgCl). This type of electrode continues to dominate the commercial market due to its superior properties, such as high biocompatibility, electrochemical stability, and optimal electrical conductivity, which are essential characteristics for obtaining high-quality EEG signals [26]. “AgCl is a readily soluble salt that rapidly saturates the skin and forms a stable electrode–leaf interface”, according to [27]. In terms of utilization, the Ag/AgCl electrode is often paired, to reduce the electrode–foot impedance for a high-quality EEG signal, with a conductive gel.

Intra-auricular EEG solves some of the limitations associated with traditional wet electrodes, such as the discomfort caused by prolonged use and the challenge of maintaining stable contact between the electrode and scalp. The use of wet electrodes primarily involves scalp preparation that may even include hair trimming, skin decontamination, and the application of conductive gel, all time-consuming procedures that can affect the recording process. Another disadvantage is the limitations associated with long-term use; due to dehydration and coagulation of the gel, there is also progressive degradation of EEG signal quality. Conductive gel can also cause biocompatibility problems, resulting in skin irritation and even allergic reactions in some patients. Current research is focused on the development of advanced electrodes to minimize these limitations and improve the performance and comfort of EEG systems [28].

**Figure 8:**
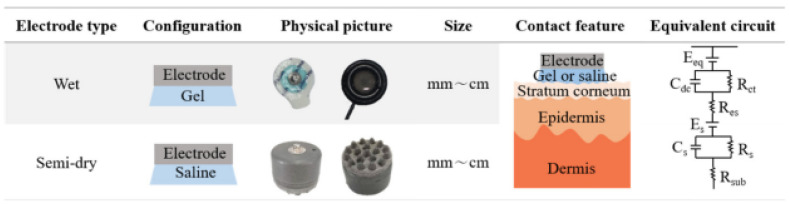
Wet and semi-dry electrode configurations. Taken from [28].

Wet electrodes consist of a disk made of and/or coated with silver/silver chloride (Ag/AgCl) in combination with conductive gel (Figure 8). This type of EEG signal collection ensures a stable connection between the electrode and the skin with the conductive gel layer. The electrolyte used has water, saline, or phosphate-buffered saline (PBS) in its composition and shows an increased tendency to detach from the skin, affecting the stability of the electrode contact with the scalp. Depending on the amount of liquid used, these electrodes are classified as semi-dry or quasi-dry, offering a compromise between increased conductivity and reduced reliance on traditional gels [29].

Currently, technologies are focusing on improving electrode configurations and integrating machine learning algorithms to increase the effectiveness of signal analysis. As proof, in-ear EEG technology is becoming more and more used and efficient at the same time.

#### 5.1.2. Dry Electrodes

The latest developments are based on dry contact electrodes, which are not uncomfortable for patients and additionally improve signal acquisition by minimizing noise interference and artifacts during brain activity monitoring. Dry electrodes represent an emerging trend in the development of portable EEG systems due to their ability to eliminate the need for conductive gels and simplify the process of application. They can be made of rigid or flexible conductive materials, such as gold, silver, or functionalized polymers (e.g., PEDOT:PSS), and can be directly integrated into custom intra-auricular holders.

Advantages of dry electrodes include reduced setup time, minimal discomfort, compatibility with long-term use and reusability without intensive cleaning. They are also ideal for integration into discrete and stand-alone devices. However, dry electrodes generally exhibit higher contact impedance than wet electrodes, which can negatively affect the signal-to-noise ratio and overall EEG signal quality, especially under dynamic conditions. Their performance is dependent on mechanical contact pressure and positioning stability, and signals may be more vulnerable to movement, sweating or individual anatomical variations. Table 4 summarizes the main characteristics of dry and wet electrodes used in in-ear EEG systems.

Current technological developments allow dry electrodes to be fabricated by 3D printing technology and subsequently coated with a silver or silver chloride layer to improve conductivity and electrochemical stability. In addition, advances in additive manufacturing facilitate real-time or near-real-time customization of electrode parameters, tailoring them to the specific requirements of each user or application.

**Figure 9:**
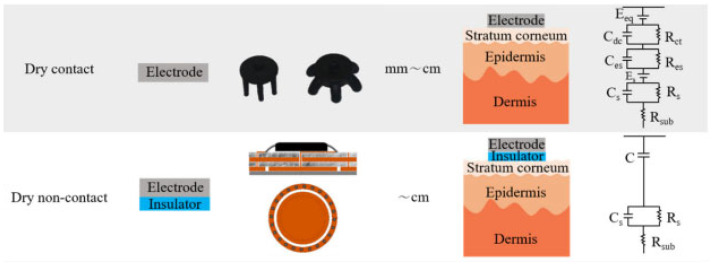
Dry contact and non-contact electrode configurations. Taken from [28].

Figure 9 illustrates the electrode design, which, although starting from a standard configuration, offers multiple degrees of freedom for adjustment and optimization.

**Figure 10:**
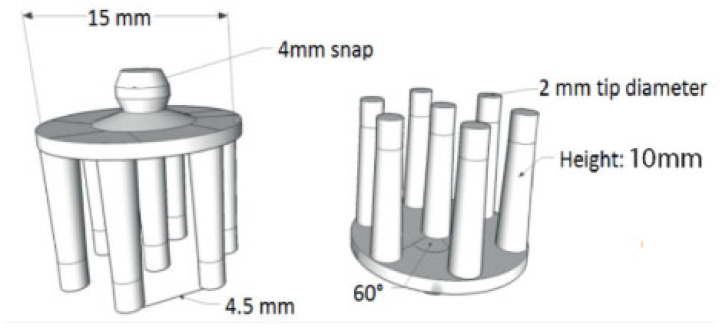
Customization parameters in EEG electrodes for realizing a better connection. Adapted from [31].

The various factors presented in the schematic influence the overall performance of the electrode, but the most important determining parameter remains the type and quality of the skin contact area. This flexibility in design allows us to improve the EEG signal, reduce the electrode–foot impedance, and increase user comfort (Figure 10) [30].

The electrode–skin interface significantly influences the performance of EEG systems; therefore, dry electrodes are recommended due to their comfort and portability [3]. A drawback of these electrodes would be the high impedance at the electrode–skin interface, which may slightly affect the quality of the recorded signal. Studies show that the impedance maintained by wet electrodes is between 10 and 20 kΩ, while dry electrodes can reach values between 200 and 1000 kΩ [5]. This aspect can lead to a reduction in the signal-to-noise ratio but requires comparative recordings with traditional EEG for the certainty of the correctness of the signal obtained in the ear.

### 5.2. Types of Electrode Placement

Electrode placement directly and significantly influences brain signal quality. In-ear EEG is used with electrodes placed in or around the ear, which provides the user with increased comfort. For a standardized electrode placement, some 10–20 or 10–10 systems have been established; the name comes from the distances between the electrodes, which are based on distances of 10% and 20% of the total skull circumference. Key anatomical landmarks, such as the nasion (bridge of the nose) and the inion (bony bump at the back of the skull), serve as reference points for these measurements. In-ear EEG refers to the recording of electroencephalogram signals using electrodes placed in the ear, mainly in the auditory canal. This innovative approach takes advantage of the proximity of the external ear to the brain, allowing a non-invasive and effective method of monitoring brain activity. Various studies have shown that EEG signals captured from the auditory canal can closely resemble those obtained from traditional scalp electrodes located close to the ear, both during cognitive tasks and during sleep states. Specifically, research has demonstrated a high coherence between EEG electrodes in the ear and standard scalp electrodes such as the T7-M1 electrode in the International 10–20 placement system, indicating shared activity between the temporal lobe and ear locations.

**Figure 11:**
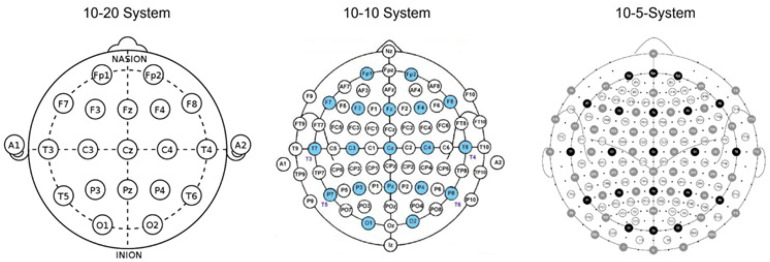
The 10–20, 10–10, and 10–5 EEG electrode placement systems, respectively.

Beyond the traditional 10–20 system, higher resolution systems have been developed, such as the 10–10 and 10–5 systems. These systems introduce additional electrodes, providing a denser range of measurements, allowing more detailed mapping of brain activity. The 10–10 system places electrodes at 10% intervals, while the 10–5 system places them at 5% intervals, enhancing neurophysiology research capabilities and improving the granularity of EEG data collected during different studies (Figure 11).

In terms of the method of electrode placement in intra-auricular EEG systems, there are fixed electrode systems—in which the position of the electrodes is predetermined and cannot be adjusted—and customizable electrode systems, which allow the electrode position to be adjusted according to the particular anatomy of the user. Fixed-electrode EEG systems are distinguished by their predefined electrode positioning, which significantly optimizes the time needed for electrode preparation and placement compared to customizable systems. This standardized configuration reduces the need for manual adjustments during setup, ensuring fast and consistent electrode application. As a result, these systems are easier to use, especially by people without extensive technical experience, thus facilitating the deployment of EEG in clinical and research contexts.

Although some studies mention the possibility of adjusting the position of the electrodes by the operator, in practice, most customized in-ear systems require a significantly more complex pre-preparation process. Specifically, customization to the individual anatomy of the ear canal requires 3D scanning and the fabrication by printing or molding of customized housings in which the electrodes are integrated. Therefore, it is not a simple manual adjustment at the time of fitting but a unique customization process that needs to be performed prior to the recording session. This step does not affect the setup time during actual use but influences the initial setup time and requires additional resources for each new user.

### 5.3. Factors Influencing Electrode Placement

Studies suggest that intra-auricular electrodes can provide high sensitivity in detecting neurological events such as seizures, thus demonstrating considerable potential for use in monitoring brain activity. The acquisition of a qualitative EEG signal depends on the correct placement of the electrodes in the ear. Thus, the optimal locations for good recording are anatomical structures of the outer ear, such as the cymbial cones and the auditory canal—areas near the temporal lobule, a brain region active in many neurological and cognitive functions [4]. The external auditory canal, from the pinna to the eardrum, is generally 26 mm long and 7 mm in diameter. These dimensions have been considered in the design of ear canal phones. In [2], the designed device does not penetrate more than 10 mm into the ear, does not localize in the bone surrounding part of the ear canal, and does not affect the eardrum in any way. In addition, the specific anatomy of the ear may help to reduce signal attenuation compared to traditional scalp-based EEG setups, thus improving the fidelity and stability of the recordings [4]. A precise localization of electrodes is of great importance in ensuring consistency of EEG signals. Usually, preauricular points are used as anatomical landmarks for electrode fixation, but there is also the possibility that their identification may be difficult due to morphological differences in each individual. Recent advances in the field of photogrammetry have led to a significant improvement in the process of localization of electrode placement points by accurately determining the anatomical areas of interest, for example, the helix–tragus junction [32]. Thus, the increased precision in electrode placement achieved with this innovative method further contributes to optimized signal collection and increased reliability of intra-auricular EEG systems [32].

### 5.4. Electrode Configuration and Mounting Techniques

The performance of in-ear EEG systems is primarily supported by the electrode configuration techniques used. Thus, a direct and major influence on the quality of the recorded EEG signal is provided by the electrode configuration, which determines parameters such as signal-to-noise ratio, artifact filtering capacity, and recording stability. Looking also from the user’s perspective, the type of electrodes used and the way they are placed also influence the patient’s experience, who may or may not be willing to wear the device for long periods of time. Factors such as contact pressure, electrode material, and method of attachment are fundamental to ensuring valid and non-intrusive monitoring. The most recent advances in the implementation of in-ear EEG devices aim to integrate dry contact electrodes. This novel approach was proposed by Looney et al. in 2011 and subsequently validated by a series of experimental studies, which support its veracity for the purpose of monitoring electroencephalographic activity under different conditions, including wakefulness and sleep [2,5]. The reliability of intra-auricular dry electrode EEG systems has been demonstrated by correlating the signals obtained with those recorded by conventional EEG methods, thus strengthening the potential of this technology for clinical and research applications in neuroscience and long-term neurological monitoring.

In-ear EEG wearable devices (bipolar mounts) are commonly used to measure voltage differences between adjacent electrodes. This configuration allows detection of local brain activity and improves the signal-to-noise ratio by reducing the influence of external artifacts. The use of bipolar montages is particularly valuable in identifying focused brain activity, facilitating the analysis of EEG signals associated with specific cognitive functions or neurological states [33]. At the opposite pole is the average reference montage, which involves using the average signal obtained from all electrodes as the reference point for analyzing EEG data. This configuration offers several advantages over bipolar setups, including reducing the influence of local signal variations. The signal-to-noise ratio is improved by attenuating common noise components. In addition, the average reference fitting helps to identify and eliminate artifacts. By comparing the obtained signals with normative databases, this method facilitates background noise filtering and allows a more robust interpretation of brain activity [34].

## 6. Signal Quality Metrics in In-Ear EEG: SNR and CMRR

For in-ear EEG recordings to have clinical and experimental credibility, their performance must be evaluated in objective metrics. This section provides a general comparison of signal quality parameters between scalp and in-ear EEG, focusing on SNR and CMRR.

**Figure 12:**
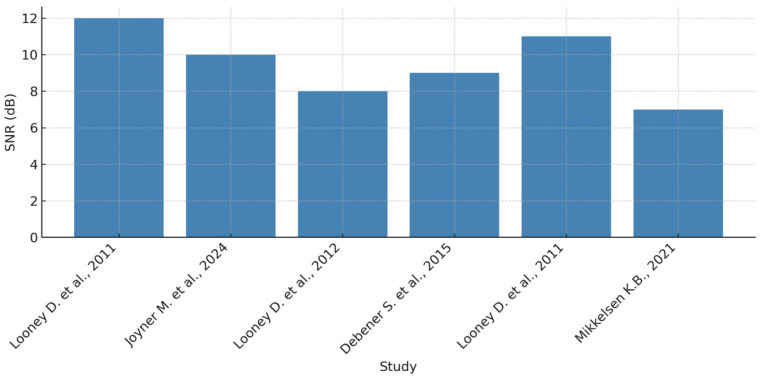
Distribution of SNR values reported in the studies by Looney et al. (2011) [2], Joyner et al. (2024) [6], Looney et al. (2012) [8], Debener et al. (2015) [10], Looney et al. (2011) [11], and Mikkelsen (2021) [12].

Signal-to-noise ratio (SNR) and common mode rejection ratio (CMRR) have been introduced. The remarkable potential of in-ear EEG technology is in direct proportion to the challenges that such a large-scale technology imposes. The differences in signal amplitudes compared to scalp EEG have generated debate about the effectiveness of in-ear EEG systems in capturing certain brain waveforms. For example, resting-state alpha waves recorded by intra-ear electrodes exhibit lower mean amplitudes compared to those obtained by scalp EEG, although the overall waveform patterns remain similar. These findings call for further investigation into the limitations and advantages of intra-auricular EEG systems, influencing both their adoption in research and clinical settings. Thus, a thorough understanding of the relationship between SNR and CMRR in intra-auricular EEG underpins technology optimization and increases the accuracy of EEG monitoring. Continuing innovations in signal processing and hardware design will help to overcome existing challenges, thus paving the way towards more efficient, affordable, and user-friendly EEG solutions with broad applications in neuroscience, medicine, and brain–computer interfaces (BCI). To highlight the key differences between conventional EEG and intra-auricular EEG, the relevant parameters were organized according to four categories: electrode type, acquisition method, signal quality, and overall system ergonomics. Table 5 provides a systematic comparison to help understand the technical tradeoffs and relative advantages of each technology. As illustrated in Figure 12, the distribution of SNR values emphasizes the variability between scalp and in-ear EEG systems.

Analysis of this table highlights that conventional EEG offers superior performance in terms of signal quality (SNR and amplitude) and is ideal for clinical applications requiring precision. However, in-ear EEG is characterized by increased comfort, portability, and usability in natural environments, which are essential for ambulatory or long-term applications. In addition, the transition from wet to dry electrodes, although involving a partial loss of signal fidelity, brings a significant gain in ergonomics and adoptability. This comparison emphasizes the need for a balance between technological fidelity and practical usability.

**Figure 13:**
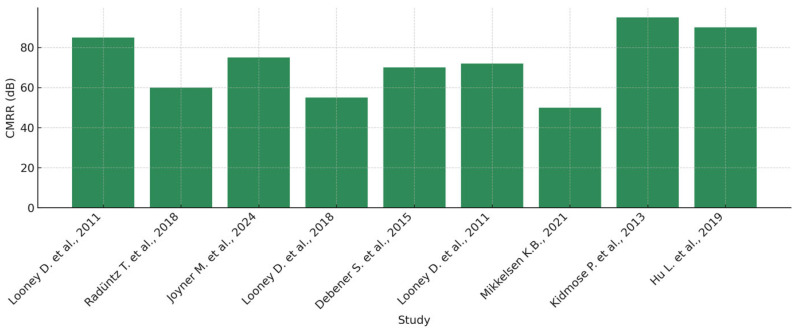
Distribution of common-mode rejection ratio (CMRR) values reported in the studies by Looney D. et al. (2011) [2], Radüntz T. et al. (2018) [4], Joyner M. et al. (2024) [6], Looney D. et al. (2018) [8], Debener S. et al. (2015) [10], Looney D. et al. (2011) [11], Mikkelsen K.B. et al. (2021) [12], Kidmose P. et al. (2013) [35], and Hu L. et al. (2019) [36].

Similarly, the CMRR level, fundamental for background noise suppression, is higher in systems using wet electrodes, as can be seen in Figure 13. It is noticeable that in-ear EEG systems with wet electrodes tend to achieve a medium to high level of common-mode noise rejection, whereas systems based on dry electrodes are more susceptible to artifacts.

## 7. Electrode Performance and Its Impact on SNR

The specific acquisition means of intra-auricular EEG and the validity of the type of electrodes used are evaluated by means of the SNR parameter. In this way, by analyzing the different SNR values, a comparison between the different EEG recording methods can be made, and it can be determined which one best meets the clinical requirements. Here, we analyze how different electrode types and materials impact the SNR values in in-ear EEG recordings.

Ag/AgCl wet electrodes have a few advantages that strongly balance the disadvantages. In terms of SNR, this type of electrode ranks first, as it offers the best SNR, with values of 10–20 dB. The conductive gel ensures stable skin contact and low impedance. Fundamental studies on in-ear EEG [2,35] categorically validate this category of electrodes because, compared to the EEG signal obtained by conventional methods, the EEG signal obtained with wet electrodes has similar values. At the opposite pole, among the most important disadvantages of wet electrodes are the need for constant gel refreshing and the long-term discomfort caused by the gel.

In antithesis, from a comfort point of view, dry electrodes gain ground over wet electrodes, but the major drawback of these electrodes is the lower SNR values, which reach values between 5 and 10 dB. The use of dry electrodes implies an increase in the variability of the recorded signal because this category of electrodes is more susceptible to motion artifacts and impedance variations. Studies in [4,5,12] have highlighted these limitations, emphasizing that wearable EEG based on dry electrodes requires advanced filtering algorithms to improve signal quality.

Printed flexible electrodes are a promising approach for EEG recordings as they incorporate advantages of both types of electrodes discussed above, providing SNRs of approximately 8–12 dB. For example, ref. [10] suggests that these electrodes propose a solution of interest for EEG monitoring in mobile contexts due to their ability to adapt to the anatomy of the ear and reduce motion artifacts. The signal-to-noise ratio also varies depending on the acquisition method used. Conventional EEG systems, based on wet scalp electrodes, achieve SNRs above 20 dB and a high common-mode rejection coefficient, providing efficient noise filtering. In contrast, wet-electrode in-ear EEGs provide SNR between 10 and 20 dB, representing a compromise between performance and portability. Models based on dry electrodes or integrated in wireless standalone devices have a lower SNR of between 5 and 10 dB, requiring optimizations to ensure higher measurement reliability.

Several aspects influence the usable signal-to-noise ratio in in-ear EEG, among which electrode-to-skin contact plays a notable role, and wet electrodes demonstrate superior stability over dry electrodes. Motion artifacts are more pronounced with dry electrodes, requiring additional algorithms for signal correction. The data processing method can contribute significantly to SNR improvement, as shown by [36], who show that advanced filtering techniques can increase the SNR by 2–5 dB.

Thus, wet electrodes remain the gold standard for EEG signal quality but are less practical for use in portable devices. Dry electrodes offer more flexibility, but at the cost of increased noise and artifacts, necessitating improved signal processing methods. Flexible printed electrodes are a promising direction that could balance performance and convenience. Also, standalone and wireless EEG systems require optimizations to ensure adequate SNR under real-world use. Future research should focus on the integration of novel materials with improved electroconductive properties and the development of more efficient filtering algorithms to maximize EEG signal quality in the ear.

## 8. In-Ear EEG Integration into Wearable Device Applications

**Figure 14:**
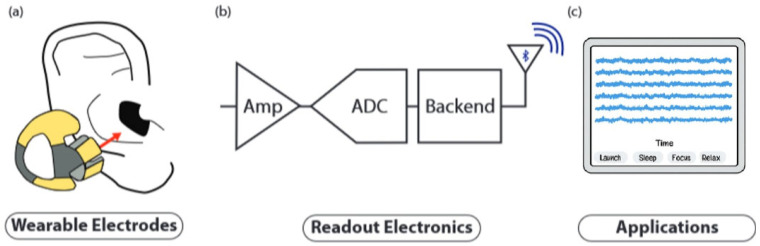
Block diagram of the generic portable system: (**a**) wearable electrodes placed inside the concha and ear canal; the red arrow indicates the signal flow direction. Different electrode colors represent distinct channels or electrode types. (**b**) Readout electronics consisting of an amplifier (Amp), analog-to-digital converter (ADC), and wireless backend for signal transmission. (**c**) Applications displaying EEG signal traces over time, labeled with example mental states such as Launch, Sleep, Focus, and Relax. Taken from [37].

The evolution of the in-ear EEG concept has led to the development of portable and wireless EEG systems, characterized by a miniaturized and esthetically pleasing design, which integrate the electrodes into discrete devices placed directly on and/or in the ear [37]. These innovative systems allow electroencephalogram recording in ambulatory mode, providing a high degree of user comfort and allowing unrestricted daily activities. In general, the block diagram of a generic portable system is shown in Figure 14.

A study, ref. [38], describes an extensive set of experimental tests carried out to validate such a portable device, demonstrating its effectiveness in monitoring brain activity under real-world conditions. In order to standardize and improve the performance of in-ear EEG devices, the authors propose a validation toolkit composed of two essential components: the EaR-P Lab version 1.0., a software application for EEG control and testing based on nine standardized paradigms, including auditory and visual evoked responses, P300 potentials, and electro-oculographic (EOG) measurements; and the Phantom ear EEG, a physical model obtained by 3D scanning of subjects’ ears and 3D printing, intended for controlled testing of different electrode configurations. The developed toolkit allowed the identification of optimal positions for the electrodes inside the ear, resulting in improved EEG responses for the auditory steady-state response (ASSR). In addition, the application of conductive paste was shown to significantly reduce impedance and background noise, increasing signal clarity. These advances open new perspectives for the use of intra-auricular EEG in neurology, telemedicine, and brain–computer interfaces, facilitating continuous recordings without the need for bulky or intrusive equipment.

### 8.1. Clinical Applications of In-Ear EEG: A Thematic Summary

In-ear EEG technology has been intensively explored over the last decade for a variety of clinical applications due to its advantages of portability, convenience, and potential for monitoring in natural environments. Clinical applications can be categorized into four main areas: sleep disorders, epilepsy, vigilance monitoring, and neurodegenerative disease detection.

#### 8.1.1. Monitoring Sleep and Improving Sleep Quality

In-ear EEG allows the recording of brain activity during sleep without disrupting the nocturnal routine and is used to identify sleep stages (NREM, REM) and fragmentation. Studies have shown that, although the signal amplitude is reduced compared to scalp EEG, in-ear EEG signals are sufficient for sleep scoring and detection of relevant events [39]. One innovative strategy is to use sleep-synchronized auditory stimulations such as tones or modulated music in order to stimulate slow waves and improve sleep quality. In-ear EEG devices can function as both receivers and transmitters, providing a platform for closed-loop interventions [10].

#### 8.1.2. Epilepsy and Continuous Monitoring of Brain Activity

One of the most promising areas is outpatient seizure monitoring. Implantable in-ear EEG systems (e.g., Epiminder) can capture pre-ictal changes and contribute to the prediction of epileptic seizures. These systems are undergoing clinical validation, and preliminary results indicate a good correlation between intra-auricular recorded activity and epileptic events detected on the scalp [40]. In addition, non-invasive in-ear EEG devices can be used for prolonged monitoring, reducing reliance on traditional laboratory EEG recordings.

#### 8.1.3. Sleepiness Monitoring and Fatigue Detection

In critical areas such as transportation and occupational safety, in-ear EEG provides a discreet solution for real-time sleepiness detection. These wearable devices are capable of classifying alertness levels using machine learning algorithms trained on EEG features. For example, systems have been reported to operate for up to 40 h continuously using gold-plated dry electrodes [27].

#### 8.1.4. Early Diagnosis of Neurodegenerative Diseases

EEG changes seen in the early stages of Alzheimer’s or Parkinson’s disease can be picked up during sleep by in-ear EEG. Studies show that changes in sleep architecture and slow oscillations may act as early biomarkers. Thus, the in-ear EEG is emerging as a non-invasive and affordable method for early screening and monitoring the progression of these diseases [40].

### 8.2. In-Ear EEG in Neurorehabilitation and Brain–Computer Interfaces

The in-ear EEG system has significant potential in the field of neurorehabilitation as a promising technology for patients with severe motor impairments. Traditionally, brain–computer interfaces (BCIs) require a certain level of volitional control to allow patients to interact effectively with therapeutic devices such as virtual reality and rehabilitation robotics. However, intra-auricular EEG offers a non-invasive and more accessible method of capturing brain activity and is an adaptable solution for patients who lack residual movement due to severe neurological conditions [5,41]. By extending access to neurorehabilitation for patients with severe central or peripheral nervous system injuries, the integration of in-ear EEG in neurorehabilitation could help monitor brain activity in real time without the need for bulky scalp electrodes; control assistive technologies like exoskeletons and neuroprosthetic devices by interpreting brain signals; customize rehabilitation programs according to neural activity patterns, facilitating functional recovery; and increase access to neurorehabilitation by offering a more comfortable and portable solution.

The ability of technology to record signals pertinent for external device control is demonstrated in [42], which describes a real-world use of in-ear EEG in BCI systems for detecting hand and tongue movement intents.

### 8.3. Research Directions

In-ear EEG technology is materializing into a basic tool in cognitive neuroscience research. This type of technology makes it possible to track brain activity while the patient performs natural movements. This feature supports and extends the validity of the recordings, as it makes it possible to interpret cognitive processes in familiar contexts, strongly reflecting how the brain acts in everyday contexts. Between the motor and cognitive systems, there is a relationship of interdependence quantified in a scientific manner using mobile EEG. This process has opened new research horizons that focus on investigating the influence of movements on cognitive functions. An emerging direction is the integration of the in-ear EEG system with non-invasive autonomic therapies, such as transcranial magnetic stimulation (TMS). With its ability to provide real-time feedback on the state of brain activity, in-ear EEG can help optimize the accuracy of closed-loop TMS therapies, allowing for tailoring treatment to individual patient needs. Continuous brain response monitoring can allow dynamic adjustment of stimulation parameters and personalization of therapy for superior clinical outcomes. The treatment of depression, epilepsy, and neurodegenerative disorders is the focus of future research directions that will explore and further integrate intra-auricular EEG data with therapeutic technologies. The symbiosis between intra-auricular EEG and TMS may propose improvements in neuromodulatory interventions, enhancing the development of non-invasive and personalized therapeutic solutions [43].

**Figure 15:**
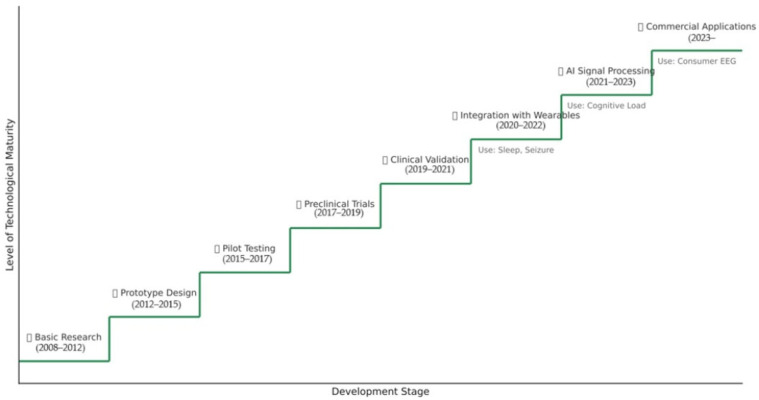
Diagram of the stage of technological development of EEG in the ear.

In order to clearly highlight the evolution and technological projections of intra-auricular EEG, the Figure 15 provides a conceptual diagram that categorizes current advances and expected directions according to the time horizon and the nature of the progress (technical optimizations vs. major breakthroughs).

## 9. Advanced Computational Techniques for In-Ear EEG

Artificial intelligence (AI) has revolutionized electroencephalography by introducing sophisticated techniques for analyzing brain activity, deep learning-based denoising algorithms, and real-time artifact removal methods. These advances enable increased accuracy in the interpretation of EEG signals, essential for diagnosing neurological disorders and improving brain–computer interfaces (BCIs) [44]. The integration of artificial intelligence into EEG technology is important for its potential to automate complex analyses, thus increasing the efficiency of clinical applications and research efforts. EEG analysis shows a continuous trend in self-improvement achievable with deep learning. Deep learning is a subset of machine learning and integrates algorithms specialized in artifact detection and reduction. Eye or muscle movements result in unavoidable physiological artifacts, but these artifacts permanently degrade the quality of EEG data. Specific deep learning algorithms succeed in minimizing the effect of artifacts with multilayer neural networks [3,4]. Traditional techniques (ICA) described in Section 4.5 have also been used to remove artifacts. However, these techniques have several drawbacks, including the constant intervention of an expert and the imminent danger of losing a large amount of data [45,46]. Deep learning techniques, on the other hand, automate this procedure and show better results in denoising and real-time applications [47].

A prominent technique in this field is dual pathway autoencoder (DPAE), which excels at separating and removing specific types of artifacts from EEG signals [47]. In addition, convolutional neural networks (CNNs) have shown great promise in processing EEG data, allowing simultaneous removal of ocular and myogenic artifacts [48]. Although CNNs are computationally demanding, recent optimizations and model compression techniques allow their use in near-real-time EEG signal processing in wearable devices. These adaptations are particularly relevant for in-ear EEG systems, where resource constraints and real-time requirements must be carefully balanced. These innovations not only increase the clarity of EEG signals but also facilitate faster processing, essential for applications requiring immediate feedback, such as in BCI [12].

In-ear EEG technology is increasingly emerging as a versatile and emerging platform for wearable neurotechnologies. Beyond its direct clinical applicability, in-ear EEG provides the prerequisites for the development of continuous brain activity monitoring systems in everyday life, fostering the transition from laboratory-controlled paradigms to user-oriented, ecological analysis.

A promising direction is the integration of artificial intelligence algorithms for real-time signal processing. Convolutional neural networks (CNNs) and Long Short-Term Memory (LSTM) models enable automatic identification of brain patterns and artifacts, optimizing accuracy in dynamic contexts. Also, federated learning can help address inter-subject variability by enabling the training of models across decentralized datasets, preserving user-specific features without requiring data centralization [23,49].

Another emerging area is the integration of in-ear EEG with neuromodulation therapies, such as transcranial magnetic stimulation (TMS), in closed-loop systems. Real-time EEG monitoring can allow automatic adjustment of stimulation parameters, leading to personalized therapies in the treatment of depression, epilepsy, or cognitive disorders. In-ear EEG systems, by their portability and unobtrusiveness, are ideal candidates for such applications [27,50].

In parallel, a need for predictive models to optimize electrode design and positioning based on individual morphological data is emerging. Integration of 3D scans, bioelectrical simulations, and machine learning methods can lead to customized electrodes that maximize comfort and signal performance. Such hybrid frameworks could significantly reduce the development and calibration time of intra-auricular EEG devices [5,7,51].

Through the convergence of miniaturization, personalization, artificial intelligence, and adaptive neuromodulation, in-ear EEG has the potential to become a pillar of future neurotechnologies. Expanding clinical trials, standardizing protocols, and developing integrated solutions will be essential to fully mature this field.

### 9.1. Deep Learning Algorithms for Denoising and Artifact Detection

AI encompasses a range of methodologies, including machine learning and deep learning. Machine learning refers to AI systems that can adapt with minimal human intervention, whereas deep learning—a subset of machine learning—utilizes multiple layers of neural networks to learn more efficiently from large datasets [4,52]. Recent reviews have highlighted the application of signal processing and machine learning techniques in EEG-based BCIs. Despite this, they often overlook deep learning, which has become a staple tool in feature engineering due to its ability to automatically identify relevant features of data without manual input [53,54].

The field of EEG analysis is making remarkable progress due to the implementation of deep learning frameworks. These frameworks not only increase the accuracy of EEG interpretation but also facilitate real-time artifact removal, thus improving the quality of EEG signals for further analysis [54]. The automatic nature of deep learning models allows for continuous improvement in the interpretation of EEG data, making them invaluable in clinical and research settings where accuracy is critical [54].

### 9.2. EEG Enhancement Techniques

EEG signals are often contaminated by various artifacts that can make analysis and interpretation difficult. Signals recorded by EEG inevitably contain artifacts that hamper the data analysis and interpretation processes.

Deep-learning methods propose, by means of specific algorithms, the automatic removal of artifacts using different signal processing techniques. Real-time processing of the obtained EEG data is performed by deep-learning algorithms to reduce the latency associated with traditional methods [55]. Traditional artifact removal methods include ICA, wavelet transform, and adaptive filtering. While deep learning automates feature extraction and artifact detection to a significant extent, expert oversight is still important for model validation, parameter tuning, and interpretation of outputs, especially in clinical applications. Simultaneously, with artifact removal also occurs the loss of significant informational layers [56,57], which is why the use of deep-learning techniques is favored for EEG signal distortion.

The main advantages of deep-learning methods consist in automating the artifact removal process without the need for specialized supervision. In this respect, convolutional neural networks (CNNs), whose architectures are specifically designed for EEG denoising, have been introduced. CNN extracts noise features using multiple convolutional blocks, subsequently reconstructing clean signals [57]. Mean squared error (MSE) and cross-correlation coefficients are parameters that objectively materialize the effectiveness of the models.

### 9.3. Deep-Learning-Based Denoising Methods

Deep-learning-based denoising techniques aim to achieve the transition from artifact-contaminated EEG signals to clean EEG signals using complex nonlinear functions and subsequently mapping the useful EEG signal. This nonlinear mapping function could distinguish between artifact and useful EEG signals [47,58]. From the initially obtained EEG signals, artifacts are separated by convolutional blocks, resulting in denoised signals. The reconstruction of these signals forms layers that finally form the denoising model [59].

EEG denoising is performed by CNNs whose architecture allows analysis and learning based on multidimensional data. CNNs hierarchically present several convolution layers preceded by pooling and then denoising layers to minimize overfitting and maximize learning [59].

### 9.4. Removal of Artifacts

The most common artifacts in intra-auricular EEG are ocular and myogenic. Thus, deep-learning methods focus particular attention on the simultaneous removal of these physiological artifacts. The CNN architectures described above are favored to be used in this context because significant improvements in denoising performance have been found to occur through their use [48,59]. Real-time artifact removal is, concretely, a goal of particular importance in improving the quality of electroencephalogram (EEG) signals.

U-Net—a specific deep-learning EEG artifact management architecture that successfully manages EEG artifacts—is worth mentioning in this respect. Currently, U-Net has been deployed in removing artifacts from electrooculograms, a reason that supports its versatility in real-time applications [60,61]. From a methodological perspective, the artifact removal process involves several steps. First, the identification of contaminated signals takes place. It then selects and decides which data segments will be kept and which will not. In systems such as BrainVision Analyzer 2, this is accomplished with tools that allow manual, automated, or semi-automated inspection of the data. The software provided assists in the data segmentation process by dividing the data into intervals that can be labeled as “Bad Interval” or discarded completely [48].

## 10. AI-Based Signal Enhancement Techniques for In-Ear EEG

Although artificial intelligence techniques are widely applied in general EEG signal processing, their role becomes particularly critical in the context of in-ear EEG. Due to the compact electrode arrangement, reduced signal amplitude, and increased susceptibility to motion artifacts, in-ear EEG systems rely heavily on advanced denoising, artifact removal, and real-time signal classification algorithms. Therefore, this section presents relevant AI-based approaches with direct applicability to in-ear EEG systems, especially in wearable and ambulatory contexts.

Neural networks that perform feature extraction using deep learning techniques [62,63] have been increasingly proposed to support or replace traditional artifact removal methods. Convolutional neural networks (CNNs) and other architectures allow the automatic identification of relevant patterns from complex EEG signals, offering improvements over classical techniques such as the short-time Fourier transform (STFT) and discrete wavelet transform (DWT) [64].

In clinical contexts such as epilepsy monitoring, the availability of clean EEG signals is critical. Automating artifact removal improves seizure detection accuracy and enhances the quality of the acquired signals. Additionally, neural networks contribute to dimensionality reduction, enabling the processing of larger EEG datasets [65,66].

Despite their potential, deep learning-based EEG methods face several challenges, including the need for large annotated datasets, risk of overfitting, and high computational demands. Furthermore, inter-subject variability and low signal resolution—particularly relevant in in-ear EEG—further complicate classification and interpretation [67,68,69].

Recent innovations, such as generative adversarial intelligence (GAI), have been explored to address these limitations. GAI models may also improve the robustness of brain–computer interface (BCI) applications, especially for users with motor impairments [70]. The ability of neural networks to enhance brain signal interpretation holds great promise not only for clinical care but also for the development of portable assistive neurotechnologies [71,72]. This is also supported by recent clinical findings by Zeydabadinezhad et al., who developed a customized in-ear EEG device. This device successfully used classical machine learning models—such as random forests and logistic regression—to classify seizures and sleep states using long-term ambulatory EEG data. Their results demonstrated high sensitivity (91%) and AUC values of up to 0.99, confirming the feasibility of combining in-ear EEG with machine learning techniques for real-world clinical monitoring [73].

### 10.1. Neural Networks

Neural networks are a subset of machine learning and serve as the fundamental architecture for deep learning algorithms. Their name reflects their design, which mimics the way the human brain transmits electrical signals through neurons [65,67]. At a high level, a neural network consists of an input layer, several hidden layers, and an output layer.

Neural networks consist of architectures on which deep learning algorithms are based. This type of network reflects, as the name itself emphasizes, the unique design that mimics the process of electrical signal transmission in the brain through neurons [65,67]. Looking deeper, a neural network consists of three layers: an input layer—responsible for receiving data. Data initially accepted at this layer can be used both for model training purposes and for testing a pre-existing model. Subsequently, several hidden layers are encountered at which the neurons perform various mathematical operations for the purpose of identifying and distinguishing specific integrated patterns. After the layers have traversed all the network structures are directed to the last layer, the output layer, which generates predictions based on previously identified patterns [68,69,74].

### 10.2. Neural Network-Based EEG Feature Extraction

In applications involving BCI and monitoring of neurological disorders, neural network-based feature extraction has emerged as a much-needed approach to be realized. CNNs underlie this method, and the congruence between the two aspects has led to increased classification performance compared to traditional techniques [71,72,75]. The goal of the research materializes in the improvement of patients’ quality of life that is possible by detecting and predicting epileptic seizures, for example. This enhanced detection is based on the neural network-based extraction process [72].

### 10.3. Advantages of Neural Network-Based EEG Feature Extraction

Neural networks, as a subset of machine learning, have demonstrated significant advantages in EEG feature extraction and classification tasks. These models excel at automatically discovering complex patterns in data, thus eliminating the need for manual feature selection, which can often lead to suboptimal results [68,76]. Their feature extraction capability is particularly enhanced in deep learning architectures, enabling efficient diagnosis of neurological impairments and disorders. Studies have indicated that deep learning algorithms provide superior classification and understanding of brain signals compared to traditional methods [68,77]. In addition, neural networks can handle complex datasets more efficiently, especially in cases where high dimensionality is present. They are adept at handling different types of data, either in the time, frequency, or spatial domain, making them versatile tools for EEG analysis [68,78]. In addition, they can utilize various strategies, such as transfer learning and data augmentation, to improve performance even when working with limited data, thus extending their applicability in various clinical scenarios [68,77].

### 10.4. Limitations of Neural Network-Based EEG Feature Extraction

Despite their advantages, neural networks also face significant limitations. A major challenge is the dependence on large datasets for efficient training. Neural networks can be prone to overfitting, especially when trained on small datasets or on datasets with few relevant data points [68,77]. Since the performance of these models is highly dependent on the quality and quantity of the data, small or poorly representative datasets may decrease the overall predictive accuracy. In addition, neural network models often require extensive computational resources and training time, making them less affordable for certain applications [68]. The complexity of hyperparameter tuning adds another level of difficulty, requiring a careful approach to ensure optimal performance. In scenarios where data sparsity is an issue, simpler classification methods, such as naïve Bayes or short decision trees, may be more effective due to reduced susceptibility to overfitting [68,77].

### 10.5. Performance Metrics

Evaluating the performance of neural network models used in the extraction of electroencephalogram features is important to ensure accurate diagnoses and effective treatment recommendations. Various performance metrics can be used, each with its specific relevance depending on the context of the study and the nature of the EEG data. Accuracy is one of the most fundamental metrics, representing the proportion of true findings (both true positive and true negative) out of all cases examined. In EEG studies, achieving high accuracy is vital for reliable diagnosis and treatment strategies [70,79].

Precision and recall are essential in scenarios where false positive and false negative costs differ significantly. Precision quantifies the accuracy of positive predictions, while recall (or sensitivity) measures the model’s ability to identify all relevant cases. These metrics are particularly important in epileptic spike detection, where missing a spike can lead to serious implications [79]. The F1 score, defined as the harmonic mean of precision and recall, provides a single measure that balances both concerns. This metric is particularly useful in unbalanced datasets, which are prevalent in EEG studies, such as when the occurrence of events such as seizures is rare compared to non-events [24,56].

Specificity measures the proportion of true negative outcomes that are accurately identified. High specificity is essential in EEG studies to minimize false alarms in clinical settings by ensuring that only genuine events are flagged for further analysis [24,79].

The area under the receiver operating characteristic curve (AUC-ROC) serves as a performance measure for classification problems at different threshold settings. It provides an aggregate measure of performance for all possible classification thresholds, which makes it a valuable measure for evaluating the trade-off between sensitivity and specificity in classifying EEG data [56,79].

The balanced F-score is an extension of the traditional F1 score, designed to handle unbalanced datasets more efficiently. This metric is particularly useful in EEG studies where classes are not equally represented, providing a more nuanced assessment of model performance [78,79].

### 10.6. Future Directions

The integration of generative artificial intelligence (GAI) and brain–computer interfaces has bidirectional benefits that could significantly improve both fields in the near future [80]. As the technological landscape evolves, it is essential to explore the potential of real-time applications that can improve the efficiency and accuracy of EEG feature extraction methods. There is an urgent need to advance real-time systems so that their accuracy rivals that of existing robust but computationally intensive methods. This transition is essential for gaining clinical acceptance of these technologies in the future [81]. By improving real-time processing capabilities, the healthcare system could alleviate some of the current stresses, particularly in terms of patient delays. Such improvements would facilitate the processing of a larger number of patients in a shorter time [81,82].

## 11. Design Optimization for Long-Term In-Ear EEG Monitoring

Intra-auricular EEG devices, the so-called in-ear EEG systems, are a state-of-the-art innovation in brain activity monitoring using compact, non-invasive sensors placed in the ear canal. These systems stand out for their potential to revolutionize brain–computer interface (BCI) technology, offering an optimal balance of convenience, portability, and discretion compared to traditional scalp EEG. The unique design of the in-ear EEG devices allows accurate neural signal capture while minimizing external interference and motion artifacts, making them a promising option for a wide range of applications, including neurorehabilitation, epileptic seizure monitoring, and early diagnosis of neurodegenerative disorders [83].

The design of EEG devices, particularly those intended for use in the ear, requires a complex and multidisciplinary approach, considering key factors, described below, that influence usability, user comfort, and the quality of EEG signal acquisition.

### 11.1. Comfort and Ergonomic Fit

One of the main characteristics that a wearable device must fulfill, even more so the in-ear EEG, is comfort during wear. Comfort and discretion are paramount in designing in-ear EEG devices, especially for long-term applications such as sleep monitoring. If the device is uncomfortable, users are less likely to tolerate it, leading to compromised data quality. Post-experiment surveys indicate that the comfort level of generic in-ear EEG is comparable to that of customized alternatives, suggesting that material choice and design significantly influence user acceptance [84].

According to studies, this component has a direct impact on user preference and technology adoption for daily use [83]. Research suggests that an optimized ergonomic design can improve device acceptability, reducing discomfort and allowing EEG monitoring for extended periods. To ensure an accurate and comfortable fit, a comprehensive approach to ergonomic design is required, based on the integration of 1D and 3D anthropometric data, which allows devices to be tailored to individual anatomical variations, and statistical shape models, which combine geometric data with anthropometric features, facilitating device customization for each user [6].

Key strategies for optimizing the comfort and usability of such devices include group-fit design, which is the creation of standardized designs that fit a wide range of users, thereby minimizing discomfort; cluster-fit, which involves optimizing the design for specific subgroups of users based on anatomical shape analysis; and, last but not least, individual-fit, which is the development of custom-fit devices shaped to the contours of each user’s ear, maximizing comfort and stability during wear [6].

### 11.2. Aesthetic Design and Non-Invasive Ergonomics in In-Ear EEG Technology

Besides, for comfort, the visual appearance of EEG devices has an important role in the user acceptance and adoption of technology. There are research studies that imply that even though comfort is the most important and dominant factor in user preference for most EEG devices, the esthetic qualities have the most significant influence in some cases. For example, in the case of the EPOC device, visual design was found to be more important than comfort, demonstrating the strong impact of esthetic factors on user perception [40]. The three-tiered model of emotional design emphasizes the importance of creating products that generate positive emotional responses, influencing not only usability but also users’ esthetic perception and self-image. Thus, an in-ear EEG device must not only be functional and comfortable but also visually appealing to enhance the user experience and facilitate its integration into everyday use.

To improve user acceptability and user experience, designers should consider aspects such as the materials used and premium finishes, suggesting quality and innovation, but also a discreet and modern design adapted to the user’s lifestyle. Customizable elements can also be integrated, such as color, shape, or texture variations, for a more pleasant experience. By harmonizing functionality with esthetic design, in-ear EEG devices can become more appealing to a wide range of users, increasing acceptance and natural integration of the technology into everyday life [83].

### 11.3. Fundamentals of EEG Signal Processing

From a methodological point of view, the EEG signal obtained by intra-auricular systems can be processed using the same established techniques applied in conventional EEG, such as independent component analysis (ICA), principal component analysis (PCA), wavelet transforms, or artificial intelligence-based models as described above. The differences do not derive from the nature of the signal itself but from the technical characteristics of the recording system: reduced number of channels, restricted layout, and lower SNR.

These limitations affect the effectiveness of spatial methods (such as ICA or beamforming), especially in contexts requiring multiple source separation or extensive cortical mapping. However, for well-defined purposes—such as detection of drowsiness states, auditory evoked responses, or continuous monitoring—classical methods can be successfully adapted and applied in the in-ear EEG context as well.

Efficient EEG signal acquisition is essential to ensure the performance and reliability of in-ear systems. Although EEG signal processing methods—such as independent component analysis (ICA) or principal component analysis (PCA)—can be applied to both conventional and in-ear EEG systems, their efficiency is often influenced by the specific limitations of in-ear systems. In all these include reduced spatial diversity, lower signal-to-noise ratio, and increased susceptibility to motion artifacts, muscle interference, and electrode–electrode contact variations [85].

In this situation, the design process of in-ear EEG systems must gather solutions suitable to these challenges, like in the following examples: optimized electrode configurations to maximize signal quality, selection of positions with minimal impedance (e.g., cymba conchae or external auditory canal), and the use of advanced materials—including nanomaterials or conductive polymers—to improve stability and comfort in use.

Moreover, to these hardware aspects, the processing of the advanced signal techniques could be applied in order to increase the robustness of the system, and here are included noise reduction algorithms, ICA/PCA for artifact separation, machine learning models for motion and muscle activity interference detection, and methods to compensate for EEG signal nonstationarity—such as dynamic normalization, wavelet analysis, and recurrent neural networks (RNN) for real-time interpretation.

The incorporation of all these components concurs with reliable signals even under ambient conditions, backing up the validity of intra-auricular EEG systems for modern clinical and computational applications.

### 11.4. Testing and Feedback

To ensure that in-ear EEG device design options are aligned with user needs and expectations, extensive usability testing is essential. Iterative testing allows not only to validate technical requirements but also to adjust the design to optimize comfort, esthetics, and overall user experience.

Traditional testing methods are expensive and time-consuming, but modern approaches based on 3D scanning and anthropometric databases offer fast and efficient alternatives to optimize the testing process [8]. Thus, these technologies allow rapid prototyping of devices adapted to different ear canal morphologies, digital simulation of fit and comfort prior to the physical production of a model, and customization of devices to ensure an optimal and stable fit. One of the most important aspects of testing is the collection of user feedback, which helps to improve the devices from several perspectives, namely, from a comfort perspective—adjusting the design for long-term wear without discomfort; from a stability perspective—optimizing the fixation of the electrodes to prevent loss of contact; and from an esthetic and acceptability perspective—adapting the design to the user’s preferences for natural integration into everyday life [8].

## 12. Current Limitations and Challenges

Despite promising advantages, in-ear EEG technology continues to face significant challenges, particularly in terms of optimizing the balance between user comfort and EEG signal fidelity. Although in-ear EEG technology has made significant progress, a number of technical bottlenecks and controversies remain, thereby limiting its clinical and commercial applicability.

A first example worth detailing is the trade-off between electrode impedance and user comfort. Although dry electrodes are very comfortable and perfect for extended use, their high impedance has an impact on the signal-to-noise ratio (SNR). Conversely, low impedance is provided by moist or gold-plated electrodes, although they frequently have proportions that may impact hearing comfort or need conductive gels. Additionally, it is challenging to standardize electrodes and their placement due to individual differences in auditory canal anatomy. Furthermore, in the mobile setting, motion artifacts and muscle noise are more noticeable, necessitating the use of more complex processing methods. Another point of contention concerns its poor spatial resolution—intra-auricular EEG only offers localized information due to its fixed architecture in the ear region and small number of electrodes, which makes it inappropriate for applications needing extensive cortex mapping.

The security of long-term use must also be considered, necessitating the creation of strong technological solutions that reduce motion artifacts and preserve a consistent level of EEG recording quality. To improve the functionality and suitability of in-ear EEG in practical settings, these difficulties highlight the necessity of advancements in electrode design, device flexibility, and the application of cutting-edge signal processing algorithms.

Despite research demonstrating that in-ear EEG enables efficient brain function monitoring, including sleep staging and epileptic activity identification, this method still has drawbacks. The primary issues are signal quality, which is impacted by the high impedance at the electrode–lead interface, and motion artifact susceptibility, which is still up for discussion and investigation.

## 13. Perspectives and Predictive Models

It is becoming clear that integrated predictive frameworks are required to enable the optimal design of in-ear electrodes in order to avoid the present drawbacks of in-the-ear EEG technology. A model like this might integrate bioelectric–mechanical simulations that correlate metrics like contact pressure, interface impedance, and estimated comfort level with specific anatomical modeling based on 3D scans of the ear canal.

Additionally, EEG signal performance could be anticipated because of individual differences in electrode material, placement arrangement, and ear shape by incorporating machine learning techniques. This kind of framework would enable the creation of specialized solutions where the functional properties and physical design of the electrodes are jointly adjusted to provide a balance between user comfort, mechanical stability, and EEG recording fidelity. By using these predictive models, clinical applications could move more quickly from experimental prototypes to reliable and portable ones.

The dual challenges of optimizing EEG data quality and guaranteeing user comfort are the focus of in-ear EEG technology development efforts in terms of future research paths. For the devices to be worn for extended periods of time without causing discomfort or compromising signal stability, the ergonomics of the in-ear EEG are an important consideration in this regard.

Important elements, including the electrode location, materials used, and overall device design, have a direct impact on the device’s capacity to adjust to the unique anatomy of each user and reduce motion artifacts and impedance. Future in-ear EEG systems may increase the accuracy of EEG recordings by including biocompatible materials, flexible constructions, and optimal electrode designs. This would increase the technology’s suitability for long-term neurophysiological monitoring.

## 14. Towards Safe and Ethical In-Ear EEG Monitoring: Long-Term Biocompatibility Challenges

Sound ethical frameworks must be developed to handle the difficulties with long-term biocompatibility as EEG technology advances, particularly when it comes to implanting devices in delicate areas like the ear. The variability of electrode settings, together with individual anatomical particularities, can significantly influence signal quality, generating potential discrepancies between data obtained by intra-atrial EEG recordings and those obtained by conventional scalp methods [86]. In addition, the potential for tissue reactions and the safety of the materials from which electrodes are made for long-term applications raise ethical questions of notable importance, targeting the health of the user and the need for informed consent in the context of medical device research and deployment [86].

Regulatory considerations regarding the use of EEG in the ear thus emphasize the need for rigorous ethical standards. Adherence to safety rules and protection of the privacy of personal data are essential prerequisites for guaranteeing users’ rights and for responsible deployment of these technologies [71,87,88].

### 14.1. Ethical Aspects

The ethical considerations related to the development and implementation of technologies, particularly in medical and scientific contexts, are multifaceted. Of major interest, for example, are the ethical implications of the communication and consent process in research involving people with severe speech difficulties, such as individuals diagnosed with locked-in syndrome (LIS). These patients, although fully conscious, are almost completely paralyzed and depend on alternative methods, such as eye tracking or residual muscle twitching, to express their thoughts and emotions [89,90].

One fundamental component of ethical research conduct is consent. However, obtaining informed consent can be quite difficult for someone with poor communication skills. Since participants must be able to speak effectively to give their consent to engage in studies, there are significant ethical concerns regarding how consent may be appropriately obtained in these situations [89,91]. Bauby’s ability to compose a book with just his left eye blinking serves as an example of this battle, emphasizing both the possibility of communication in challenging circumstances and the significance of encouraging these communication techniques in study environments [89]. Adopting a relational ethical framework—which emphasizes the relationships between participants and researchers rather than only risk-based approaches—may be necessary to address these ethical conundrums [5]. In order to facilitate communication and obtain permission, this viewpoint places a strong emphasis on comprehending the participant’s background and building mutual respect and trust. To guarantee the methodical integration of ethical issues into the research process, it is crucial to incorporate ethical design standards, such as those described in IEEE Std 7000™-2021 [92]. These guidelines offer ethical design techniques, encouraging an all-encompassing strategy that upholds the rights and dignity of every participant.

### 14.2. Biocompatibility of In-Ear EEG Devices

The use of implantable medical devices, such as, in this case, the in-ear EEG, requires compliance with a few rules and issues related to long-term biocompatibility. Biocompatibility is the ability of biological systems to interact with material without the material generating a dangerous immune response. The materials used in the manufacture of medical devices are subjected to a series of biocompatibility tests covering chemical, mechanical, and biological characterization. All these properties follow guidelines previously established by the International Organization for Standardization (ISO) 10993 [93,94].

In-ear EEG involves, as the name emphasizes, the insertion of a device containing specialized electrodes inside the ear, i.e., at the level of the cochlea. Placement of the earphone inside the ear canal can cause an inflammatory response as the body reacts to the presence of a foreign body, ultimately leading to tissue growth around the device. According to studies, one example of this is silicone, which is a biomaterial often used in in-ear EEG systems that can trigger immune or inflammatory responses [92,93]. The inflammatory responses that have occurred have been shown in histological analysis to be the presence of immune cells such as lymphocytes and macrophages in samples taken from around the electrodes [92,94]. At the same time, previous research has also validated the lack of toxicity of certain silicone compounds, so that the inflammatory reaction should not be viewed strictly in terms of the cytotoxicity of the material itself but is primarily a reaction of the biological system to the presence of a foreign body [94,95].

In support of maximizing the longevity and performance of implantable devices, the aim has been to reduce the severity of the inflammatory reaction. Thus, by administering anti-inflammatory drugs (e.g., dexamethasone), inflammatory markers were reduced, thus improving tissue response in experimental models. This was one of many strategies proposed to address biocompatibility issues [93].

Biocompatibility is supported by the implementation of wearable EEG devices, which involves going through a series of steps. The design and selection of materials to be used are key moments in the process of supporting biocompatibility. Naox Technologies has developed an in-ear EEG system incorporating electrodes made of silicon coated with conductive silver ink. These electrodes were optimized for both biocompatibility and electrical conductivity considerations [96]. Potential immune interactions with biological tissues were minimized by using a cross-electrode configuration in the ear. Minimizing immune interactions leads, in contrast, to maximizing the performance and acceptance of the device by the body over long periods of time.

The goal of medical innovations in the context of neurotechnology is ultimately the well-being of the users. Thus, innovations in the field of EEG applications and the ethical implications of their use must be in a permanent balance. This takes the form of a continuous dialog within the scientific and medical communities to highlight the development of complex and clear ethical guidelines.

## Figures and Tables

**Table 1:** Conceptual classification.

Term	Description	Comments
In-ear EEG	It refers to the recording of brain activity by electrodes placed inside the external auditory canal in direct contact with the skin. Typically, these electrodes are integrated into earplug-like devices. This is the main approach investigated in the present work.	Electrodes are integrated into earplug-like devices. It is the main approach analyzed in the paper.
Periauricular EEG	It includes systems that use electrodes placed around the ear (behind the pinna or on the earlobe) without penetrating the ear canal.	Used as a complementary reference in this review, particularly for design and clinical applications.
Extended atrial EEG	Integrates hybrid systems that combine intra-auricular and periauricular electrodes to extend the signal detection zone, thus achieving wider coverage.	It covers a wider spectrum of detection but is not the focus of this paper.

**Table 2:** Main differences between conventional EEG and in-ear EEG.

Feature	Conventional EEG	In-Ear EEG
Number of electrodes	8–256	2–6
Position	On scalp (10–20 system)	Intra-auricular/periauricular
Signal quality (SNR)	10–20 dB	5–10 dB
EEG signal amplitude	10–100 μV	1–10 μV
Comfort	Reduced (gel, cables)	High (portable, discreet)
Movement artifacts	Lows in the lab	Possible in real life
Installation	Complex requires staff	Simplified, possibly automated
Aim	Clinical diagnosis	Naturalistic monitoring
Limitations	Reduced mobility	Lower resolution and SNR

**Table 3:** Qualitative comparison between conventional EEG and in-ear EEG across functional parameters. The comparison is based on literature-reported trends and expert assessments, using symbolic indicators (++ = high, + = medium, − = low).

Parameter	Conventional EEG	In-Ear EEG
Number of Electrodes	++	−
Placement	++	+
SNR	++	+
Signal Amplitude	++	+
Comfort	+	++
Motion Artifacts	++	+
Installation	−	++
Portability	−	++

**Table 4:** Comparison between dry and wet electrodes for in-ear EEG.

Feature	Wet Electrodes	Dry Electrodes
Impedance	Low (1–10 kΩ)	High (>100 kΩ)
Signal quality (SNR)	Retrieved	Medium–low (sensitive to movement)
Comfort	Low (gel, damp, cold feeling)	High (dry, long wear)
Application time	Longer (requires gel and cleaning)	Fast, gel-free
Reuse	Limited (gel dries)	Easy, no cleaning
Stability over time	Good in a static environment	Variable in a dynamic environment
Clinical applicability	Traditional diagnosis	Wearable systems, naturalistic monitoring

**Table 5:** In-ear EEG performance: electrode type, acquisition method, and signal acquisition parameters, respectively.

Categories	Feature	Conventional EEG	In-Ear EEG
Electrode type	Electrode type	Wet, conductive gel	Dry or hybrid (sometimes gold-plated)
	Number of electrodes	8–256	2–6
Procurement method	Position	On scalp (10–20 system)	Intra-auricular/periauricular
	Installation	Complex requires technician	Fast, sometimes self-applicable
Signal quality	SNR	10–20 dB	5–10 dB
	EEG amplitude	10–100 μV	1–10 μV
	Movement artifacts	Laboratory controllable	Present in real environments requires filtering
Ergonomics and usability	Comfort	Reduced (cables, gel)	Raised (discreet, no gel)
	Portability	Limited	Very good
	Main aim	Clinical diagnosis	Ambulatory/naturalistic monitoring
	Limitations	Stiffness, lack of mobility	Lower spatial resolution, artifacts
